# Self-Cannulation for Haemodialysis: Patient Attributes, Clinical Correlates and Self-Cannulation Predilection Models

**DOI:** 10.1371/journal.pone.0125606

**Published:** 2015-05-19

**Authors:** Anuradha Jayanti, Philip Foden, Alison Wearden, Julie Morris, Paul Brenchley, Sandip Mitra, on behalf of the BASIC-HHD study group

**Affiliations:** 1 Department of Nephrology, Central Manchester Hospitals NHS Trust, Manchester, United Kingdom; 2 Department of Biostatistics, University of Manchester, Manchester, United Kingdom; 3 Department of Psychology, University of Manchester, Manchester, United Kingdom Investigators in the BASIC-HHD study group is provided in the Acknowledgments; University of Louisville, UNITED STATES

## Abstract

**Background and Objectives:**

With emerging evidence in support of home haemodialysis (HHD), patient factors which determine uptake of the modality need to be better understood. Self-cannulation (SC) is a major step towards enabling self-care ‘in-centre’ and at home and remains the foremost barrier to its uptake. Human factors governing this aspect of HD practice are poorly understood. The aim of this study is to better understand self-cannulation preferences and factors which define them in end stage renal disease (ESRD).

**Design:**

In this multicentre study, 508 of 535 patients from predialysis (Group A: n = 222), in-centre (Group B: n = 213), and home HD (Group C: n = 100) responded to a questionnaire with 3 self-cannulation questions. Simultaneously, data on clinical, cognitive and psychosocial variables were ascertained. The primary outcome measure was ‘perceived ability to self-cannulate AV access’. Predictive models were developed using logistic regression analysis.

**Results:**

36.6% of predialysis patients (A) and 29.1% of the ‘in-centre’ haemodialysis patients (B) felt able to consider SC for HD. Technical-skills related apprehension was highest in Group B (14.4%) patients. Response to routine venepuncture and the types of SC concerns were significant predictors of perceived ability to self-cannulate. There was no significant difference in concern for pain across the groups. In multivariable regression analysis, age, education level, 3MS score, hypoalbuminemia in Groups B & C and additionally, attitude to routine phlebotomy and the nature of specific concern for self-cannulation in Groups A, B and C, are significant predictors of SC preference. The unadjusted *c*-statistics of models 1 (derived from Group A and validated on A) and 2 (derived from B+C and validated on B), are 0.76(95% CI 0.69, 0.83) and 0.80 (95% CI 0.74, 0.87) respectively.

**Conclusions:**

There is high prevalence of perceived ability to self-cannulate. Modifiable SC concerns exist in ESRD. The use of predictive models to objectively define and target education and training strategies could potentially impact on HD self-management and future uptake of home HD.

## Introduction

Haemodialysis (HD) for end stage renal disease (ESRD) remains the most widely prevalent dialysis treatment modality[1]. With mounting evidence in support of frequent and extended HD therapies, self-care HD (SCHD) and home-based HD (HHD) remain a viable and attractive option but with a low uptake, globally. Home-based treatment is associated with low technique failure[2], [3], better patient survival[3], [4], better health related quality-of-life[5][6], freedom, flexibility and employment potential[7]. HD requires access to the patient’s vascular system. Commencement of HD through a native, purposeful, surgically created AVF compared to a vascular catheter is associated with the lowest risk of death, infections and cardiovascular events[8].

Self-cannulation of AV access is an integral, yet distinct, practical component of self-care HD. Self-cannulation (SC) is the technique by which patients insert needles into their own vascular access, an AV fistula or AV graft. A haemodialysis patient is exposed to well over 300 episodes of fistula cannulation every year. In the early days, ESRD patients had to be independent for all aspects of their care including self-cannulation. With increase in numbers of well-staffed units and patient co-morbidities, there is less emphasis on self-care. The practice of SC empowers patients and creates an opportunity for them to be active participants in their own care[9]. It ensures consistent needling technique, once expert skills are attained. It allows for greater understanding of the nature of one’s vascular access including troubleshooting. With adequate education and support, best practice in vascular access care may be inculcated and practiced consistently.

Published literature in the area of self-cannulation of vascular access for HD is sparse and is largely limited to the discussion of cannulation techniques, i.e., button-hole vs. rope-ladder and associated complications such as pain[10], infections[11], [12] and vascular interventions[8], [13], [14]. Self-cannulation is the first concept; the pre-dialysis patient has to understand, for self-care HD and the first invasive step in practical application, upon commencement of home or self-care, facility-based HD training. The impact of self-cannulation concern or “fear” as a key human factor barrier to the uptake of home haemodialysis therapy has been identified in two studies[15], [16]. It poses a significant barrier to patient recruitment into these therapies both in clinical and research settings[15]. Factors which characterize the preferences for self-cannulation have not been studied in the context of ESRD.

## Objectives

This study is designed to understand self-cannulation from the patient’s perspective in a prospective multicentre study[17]. We aim to a) examine the prevalence of SC concerns in ESRD b) understand the nature of SC apprehensions c) describe the clinical and psychological correlates of these concerns d) describe the typology of predialysis or ‘in-centre’ HD patients who show SC preference and e) propose predictive models for SC preference.

To the best of our knowledge, this is the first comprehensive study of self-cannulation in a large group of patients across the ESRD spectrum encompassing predialysis patients (cannulation naive), HD patients who receive institutional care (through staff-assisted cannulation) and (self-cannulating) HHD patients.

## Materials and Methods

### BASIC-HHD study design

The study of ‘self-cannulation for HD’ is a patient factor study within a large multicenter, prospective, observational study designed to investigate the facilitators and barriers to home haemodialysis, the BASIC-HHD study (Barriers to successful implementation of care in home haemodialysis) [17]. The study involves 5 UK centres, with variable prevalence rates of home HD. An integrated mixed methodology (convergent, parallel design) has been adopted for this study in a combined cross-sectional and prospective study design. The methodological details and scope of data collected in the BASIC-HHD study has been presented in the protocol paper[17]. Broadly, these include clinical variables, neuropsychometric evaluation of participants and a compilation of questionnaires to include assessment of affect, autonomy preference and health-related quality of life. Knowledge of English language and visual intactness were required to undertake neuropsychometric tests.

### Self-Cannulation substudy population

The self-cannulation substudy data are derived from the cross-sectional segment, in which 535 patients were enrolled. Patients were enrolled from ‘predialysis’ clinics, for the CKD-5 group (Group A). Eligible patients (determined by inclusion criteria) were approached consecutively, to achieve the recruitment target at each centre. Prevalent ‘in-centre’ HD patients (Group B) were approached if they fulfilled eligibility criteria and were willing to undertake neuropsychometric assessments and complete study specific questionnaires. All self-care haemodialysis patients (93% at home) from each participating centre were approached (Group C). In all, 508 patients responded to self-cannulation questions. Demographic and clinical information was ascertained from patients and electronic medical records. Responses to self-cannulation questions from patients registered blind and disabled, were excluded from SC study analyses (n = 16). Responses to questions were recorded in an electronic database with deliberate choices. The questions on self-cannulation were posed at the same time as neuropsychometric evaluation of participants. All other questionnaires were completed by hospital dialysis patients whilst on HD, by predialysis and home HD patients in their own homes. Patients were given these to complete, at the end of their cognitive tests. The vast majority brought the completed questionnaires back at their next predialysis clinic visit (within 4 weeks) or handed them in, to the visiting renal nurse from the hospital. Patients were reminded before their scheduled clinic appointments to bring in their completed questionnaires. The patients were entrusted to complete this if they could do it independently. Where this was not feasible, a member of the research team read the questions to patients and marked patient specified responses.

### Study Registration

This study was reviewed and approved by the Greater Manchester West Health Research Authority National Research Ethics Service (NRES) Reference number: 12/NW/0170. The study is on the NIHR portfolio (ID 12346).

### Self-Cannulation questions

Three simple study-specific questions were posed to all participants (Table 1). Participants had the choice of responding to more than one answer or even describe their concerns in the free text space and appropriate categorisations were used for analysis.

**Table 1:** Questions for the self-cannulation study.

**SCQ1**	**How well do you tolerate needle insertion for blood tests?**	Permitted responses: Do not mind/Fearful/I realize it is important for my well-being
**SCQ2**	**Could you do the same (self-needle insertion), if required, for dialysis treatment?**	Permitted responses: Yes/Yes, with some help/No/Unsure
**SCQ3**	**What aspect of needling one’s self for dialysis bothers you most?**	Permitted responses: Pain/Watching the needle inserted/Fear of needle slipping out/Catastrophic bleeding/Infections/None of the above/All of the above

### Study instruments

All study participants completed a compilation of questionnaires based on measures of psychosocial factors which are perceived to be predictive of uptake of self-care HD, providing us with a quantitative measure of psychosocial state. These include the presence and extent of depression through the Beck Depression Inventory II[18]; the presence and extent of anxiety through the State and Trait Anxiety Inventory[19] and preference for autonomy through the Autonomy Preference Index[20]. Tools for objective cognitive assessment include global cognition assessment through the use of the modified mini-mental state examination (3MS)[21], and executive cognitive ability through the trail making test B (TMTB) scores[22]. The metacognition questionnaire[23] was used for subjective cognition assessment. These instruments were considered because patient’s affect and executive cognitive ability may hypothetically determine the perceived ability to undertake SC.

Some people are dispositionally more autonomous than others and may thus prefer self-care. Autonomy preference index scale used in this study was designed to measure preferences for autonomy in decision making in a general sense. The BDI is a self-report inventory that has been extensively validated and used for measuring depression in various population groups, including ESRD. Although depression in haemodialysis population is well studied, anxiety is also recognised to be a very important problem which may be present independent of other problems or somatised as part of another mental ailment. BDI (0–10, 11–15, 16–20, 21–25, 26–30, 31+), STAI (20–29, 30–39, 40–49, 50+) and 3MS (94–100:1, 86–93:2, 81–85:3, 76–80:4, ≤75:5) scores have been considered in ordered categories for analyses. 3MS categorisation is in the reverse order as mentioned above.

### Questionnaires return rate

Overall completion rate for the self-cannulation questions was 95%. The compiled validated questionnaires return rate ranged from 70%-100% for the inventories, across all participating units. The collective valid and complete responses averaged 82%.

### Statistical Analyses

Analyses were carried out using SPSS 20 and R 3.1.0. Baseline characteristics between groups were assessed using ANOVAs, chi-square tests and Kruskal-Wallis tests. The conventional two-sided 5% significance level was used. Appropriate adjustments were made to account for multiple testing when carrying out pairwise comparisons. Variables included in the analysis comprised, demographic variables, clinical parameters and psychosocial factors which are clinically meaningful in the study context. Laboratory parameters of albumin and haemoglobin are included as surrogates of physical illness.

Models for predicting those patients who would consider self-cannulation were identified using multivariable logistic regression analysis using the backward step-wise selection method. The variables of interest are shown in Table 2. In the logistic regression model, the perceived self-cannulation ability answer (SCQ2) was the outcome and age was included as a fixed covariate. Responses to SCQ2 were dichotomised as ‘Yes’ and ‘No’. The former includes- ‘Yes’, and ‘May be’ responses. Variables with p-value <0.10, were considered for selection in the multivariable logistic regression model. Any variable with significant missing data (>25%) was removed at this stage of the analysis.

**Table 2:** Demographic, clinical and psychosocial characteristics.

Characteristic		Total (N = 484)	Predialysis (Group A) N = 202	In-centre HD (Group B) N = 189	Home HD (Group C) N = 93	Overall p-value	Within group p-values
Age (Mean, SD)			58.81(13.08)	56.60(14.38)	52.16(11.79)	**p<0.001** ^**1**^	A>B p = 0.27^2^
							A>C p<0.001^2^
							B>C p = 0.03^2^
Gender	Male	313(64.7%)	125(61.9%)	120(63.5%)	68(73.1%)	p = 0.16^3^	
Education	Post high school	121(25.9%)	48(24.4%)	32(17.7%)	41(45.6%)	**p<0.001** ^**3**^	A<C p<0.05^4^
							B<C p<0.05^4^
Employment	Retired	218(45.2%)	97(48.0%)	86(45.7%)	35(38.0%)	**p<0.002** ^**3**^	
	Unemployed	120(24.9%)	39(19.3%)	62(33.0%)	19(20.7%)		**A<B** p<0.05^4^
	Self-employed	40(8.3%)	17(8.4%)	15(8.0%)	8(8.7%)		
	Salaried	104(21.6%)	49(24.3%)	25(13.3%)	30(32.6%)		**A>B** p<0.05^4^
							**C>B** p<0.05^4^
Ethnicity	Non-white	53(11.0%)	20(9.9%)	20(10.6%)	13(14.0%)	p = 0.57^3^	
Informal Care-giver	Spouse/Partner	272(57.5%)	127(63.8%)	84(45.7%)	61(67.8%)	**p<0.001** ^**3**^	**A, C>B** p<0.05^4^
	Child carer	27(5.7%)	12(6.0%)	9(4.9%)	6(6.7%)		
	Parent carer	34(7.2%)	11(5.5%)	16(8.7%)	7(7.8%)		
	Friend, Relative, Sibling or Carer	22(4.7%)	3(1.5%)	16(8.7%)	3(3.3%)		**A<B** p<0.05^4^
	Alone	118(24.9%)	46(23.1%)	59(32.1%)	13(14.4%)		**B>C** p<0.05^4^
Smoking	Never smoked	272(56.7%)	110(54.5%)	105(56.5%)	57(62.0%)	p = 0.80^3^	
	Ex- smoker	142(29.6%)	62(30.7%)	55(29.6%)	25(27.2%)		
	Current	66(13.8%)	30(14.9%)	26(14.0%)	10(10.9%)		
Self-perceived vision	Poor/suboptimal	78(16.1%)	41(20.3%)	29(15.3%)	8(8.6%)	**p = 0.04** ^**3**^	**A>C** p<0.05^4^
Diabetes	No	352(73.2%)	139(68.8%)	132(70.6%)	81(88.0%)	**p<0.005** ^**3**^	**A,B<C** p<0.05^4^
	Type 1	20(4.2%)	7(3.5%)	10(5.3%)	3(3.3%)		
	Type 2	109(22.7%)	56(27.7%)	45(24.1%)	8(8.7%)		**A,B>C** p<0.05^4^
Ischaemic Heart Disease	Yes	113(23.3%)	42(20.8%)	51(27.0%)	20(21.5%)	p = 0.32^3^	
Heart Failure	Yes	24(5.0%)	10(5.0%)	11(5.8%)	3(3.2%)	p = 0.64^3^	
Stroke	Yes	29(6.0%)	10(5.0%)	13(6.9%)	6(6.5%)	p = 0.71^3^	
Solid Organ Malignancy	Yes	53(11.0%)	18(8.9%)	16(8.5%)	19(20.4%)	**p<0.005** ^**3**^	**A, B<C** p<.05^4^
Body Mass Index	(Median, Range)		28.37(6.62, 50.78)	26.25(13.42, 49.60)	27.01(18.40, 49.96)	**p = 0.009** ^**5**^	**A>B** p = 0.02^6^
Dialysis Vintage	(Median, Range)	2.82(0, 32.98)		2.55(0, 26.98)	3.89(0.04, 32.98)	**p = 0.02** ^**1**^	**B<C p = 0.02** ^**1**^
Previous Peritoneal Dialysis	Yes	94(19.4%)	5(2.5%)	55(29.1%)	34(36.6%)	**p<0.001** ^**3**^	**A<B,C** p<0.05^4^
Previous Transplant	Yes	91(18.8%)	8(4.0%)	44(23.4%)	39(41.9%)	**p<0.001** ^**3**^	**A<B, C** p<0.05^4^
							**B<C** p<0.05^4^
Categorised BDI	0–10	224(51.5%)	96(54.9%)	84(48.3%)	44(51.2%)	p = 0.75^3^	
	11–15	72(16.6%)	26(14.9%)	31(17.8%)	15(17.4%)		
	16–20	44(10.1%)	19(10.9%)	18(10.3%)	7(8.1%)		
	21–25	41(9.4%)	16(9.1%)	18(10.3%)	7(8.1%)		
	26–30	25(5.7%)	6(3.4%)	14(8.0%)	5(5.8%)		
	≥31	29(6.7%)	12(6.9%)	9(5.2%)	8(9.3%)		
Categorised STAI-S (Anxiety State)	20–29	141(33.9%)	49(28.7%)	59(36.4%)	33(39.8%)	p = 0.52^3^	
	30–39	122(29.3%)	52(30.4%)	50(30.9%)	20(24.1%)		
	40–49	97(23.3%)	44(25.7%)	33(20.4%)	20(24.1%)		
	≥50	56(13.5%)	26(15.2%)	20(12.3%)	10(12.0%)		
Categorised STAI-T (Anxiety Trait)	20–29	111(27.1%)	42(25.1%)	44(27.7%)	25(30.1%)	p = 0.26^3^	
	30–39	120(29.3%)	42(25.1%)	54(34.0%)	24(28.9%)		
	40–49	103(25.2%)	53(31.7%)	33(20.8%)	17(20.5%)		
	≥50	75(18.3%)	30(18.0%)	28(17.6%)	17(20.5%)		
Categorised 3MS	≤75	15(3.3%)	4(2.1%)	10(5.6%)	1(1.2%)	**p = 0.02** ^**3**^	
	76–80	18(4.0%)	9(4.8%)	7(3.9%)	2(2.5%)		
	81–85	36(8.0%)	11(5.8%)	20(11.2%)	5(6.2%)		
	86–93	167(37.3%)	69(36.5%)	74(41.6%)	24(29.6%)		
	94–100	212(47.3%)	96(50.8%)	67(37.6%)	49(60.5%)		**A,C>B** p<0.05^4^
TMT B	(Median, Range)		90.0(30, 270)	108.5(39, 349)	74.5(30, 267)	**p<0.001** ^**5**^	**A<B** p = 0.008^6^
							**B>C** p<0.001^6^
Metamemory Scale	(Mean, SD)		17.92(3.70)	17.77(4.35)	17.88(3.95)	p = 0.93^1^	
Metaconcentration Scale	(Mean, SD)		14.37(2.69)	14.85(3.29)	14.77(3.13)	p = 0.27^1^	
Autonomy Preference-DM	(Mean, SD)		45.94(16.88)	52.62(17.96)	56.52(17.83)	**p<0.001** ^**1**^	**A<B** p = 0.003^2^
							**A<C** p<0.001^2^
Autonomy Preference-IS	(Mean, SD)		78.14(10.27)	82.15(11.37)	84.56(11.43)	**p<0.001** ^**1**^	**A<B** p<0.005^2^
							**A<C** p<0.001^2^
Opportunity to speak to HD patients	Yes	180(37.2%)	89(44.1%)	53(28.0%)	38(40.9%)	**p<0.003** ^**3**^	**A>B** p<0.05^4^
Haemoglobin	Hb<9g/dL	24(5.0%)	7(3.5%)	13(6.9%)	4(4.3%)	p = 0.30^3^	
Albumin	Alb< 30g/L	35(7.3%)	8(4.0%)	24(12.7%)	3(3.3%)	**p = 0.001** ^**3**^	**A,C<B** p<0.05^4^

^*1*^ *ANOVA p-value for overall between groups mean differences*

^*2*^ *Scheffe adjusted p-values for comparison of pair-wise group means*

^*3*^ *Pearson Chi-Square p-value*

^*4*^ *z-test comparing category proportions between groups*, *p-value with Bonferroni adjustment for multiple testing*

^*5*^ *Kruskal-Wallis test p-value*

^*6*^ *Mann-Whitney U test p-value with adjustment for multiple testing*

The differences in characteristics between predialysis and dialysis patient groups, led to development of two models. MODEL 1 was built using group ‘A’ data (predialysis patients) and validated on the same group. MODEL 2 was derived from data on groups ‘B’ and ‘C’ (HD patients) and validated on group B data and on the separate ‘A’ data. The predictive strength of each model was assessed using the ROC (receiver operating characteristic) curve. Discrimination was evaluated using the *c*-statistic, representing the area under the curve (AUC). Model calibration tests were also undertaken, to assess how closely the predicted probabilities reflect actual performance of the model. To adjust for overoptimism, Efron’s enhanced bootstrapping procedure was employed which allowed for internal validation of our models[24].

## Results

### Group demographic, clinical and psychosocial characteristics (Table 2)

The numbers of complete responses to the three self-cannulation questions were 491, 484 and 490 for SCQ1, SCQ2 and SCQ3 respectively.

The home haemodialysis group was younger than the predialysis and ‘in-centre’ patients. Significantly higher proportion of patients in the home group had received post high-school education. A significantly higher proportion of group B patients lived on their own. About 27% of the total study population had diabetes with significantly less diabetes in the home HD group. Approximately one-third of patients in groups B and C had a previous history of peritoneal dialysis, a home-based therapy. Significantly higher proportion of patients in group C had a previous transplant. Predialysis patients scored significantly lower than hospital and home patients on autonomy preference. In objective assessment of cognition of both memory domain and executive function, groups A and C performed better than B.

### Response to routine phlebotomy-SCQ1 (Table 3)

**Table 3:** Patient disposition towards routine phlebotomy for blood tests (SCQ1).

Response to routine venous cannulation (SCQ1)	Predialysis group (n = 203)	‘In-centre’ HD group (n = 193)	Home HD group (n = 95)	p-value (between groups)
Do not mind	148 (72.9%)	124 (64.2%)	81 (85.3%)	**p<0.001** ^**1**^
Fearful	16 (7.9%)	23 (11.9%)	2 (2.1%)	Do not mind **B<C** p<0.05^2^
Realise it is important for my well-being	39 (19.2%)	46 (23.8%)	12 (12.6%)	Fearful **B>C** p<0.05^2^

^1^Pearson Chi-Square p-value

^2^z-test comparing category proportions between groups, p-value with Bonferroni adjustment for multiple testing

The response to this question was ‘fear’ in a significantly higher proportion of group B patients who self-selected into in-centre HD with staff-assisted cannulation.

### Perceived ability to self-cannulate for HD-SCQ2 (Fig 1)

**Fig 1:**
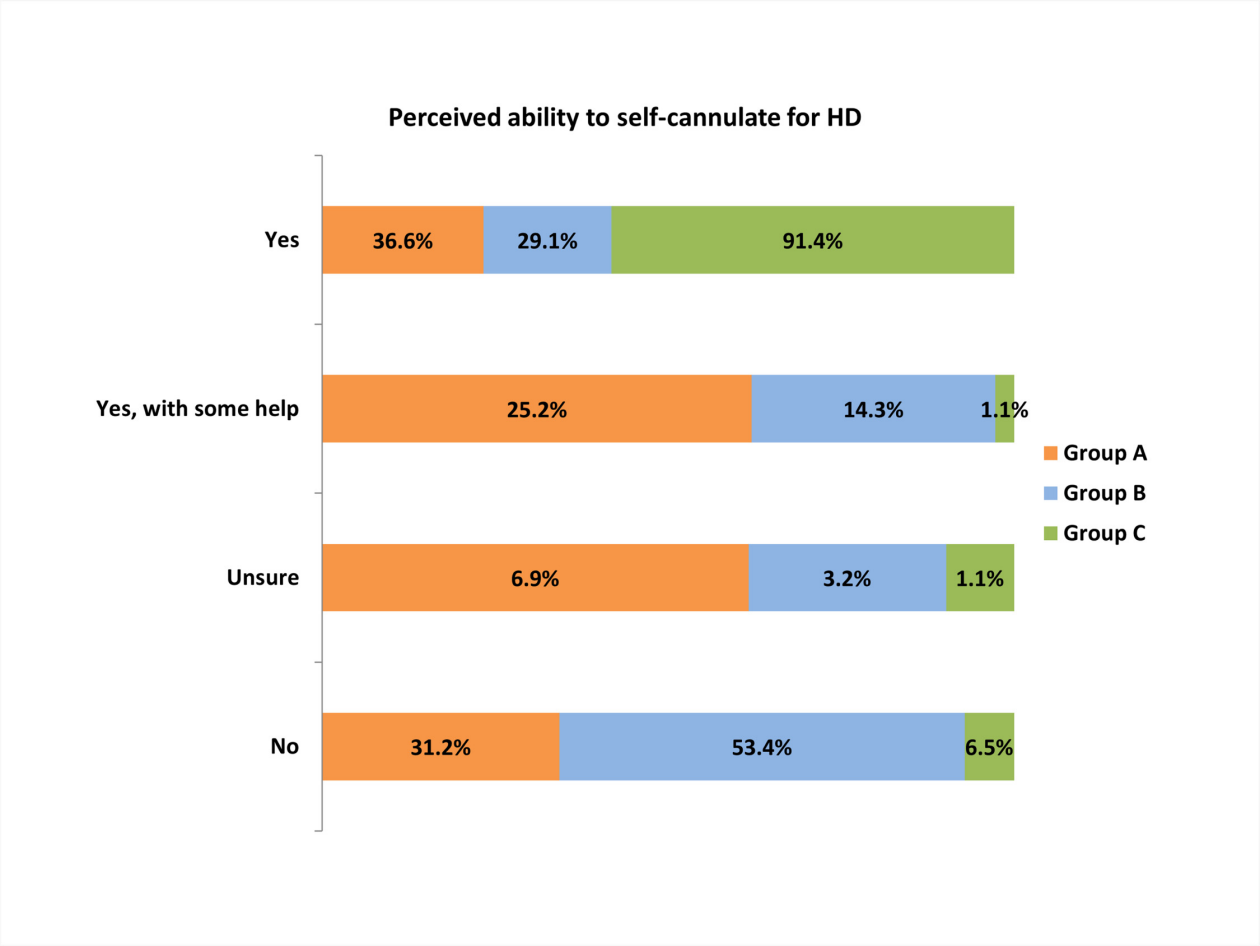
Bar chart shows the perceived ability of patients in the three study groups to self-cannulate. **Group** A: Predialysis cohort; B: Hospital haemodialysis cohort; C: Home haemodialysis cohort.

Overall 66% of responders felt they could self cannulate their AVF. A positive response was received in 36.6% in Gr A and 29.1% in Gr B. Patients who responded with a ‘No’ to SCQ2 in the self-care HD (Gr C), typically received assistance in cannulation from their informal care-givers.

### Nature of concerns-SCQ3 (Fig 2)

**Fig 2:**
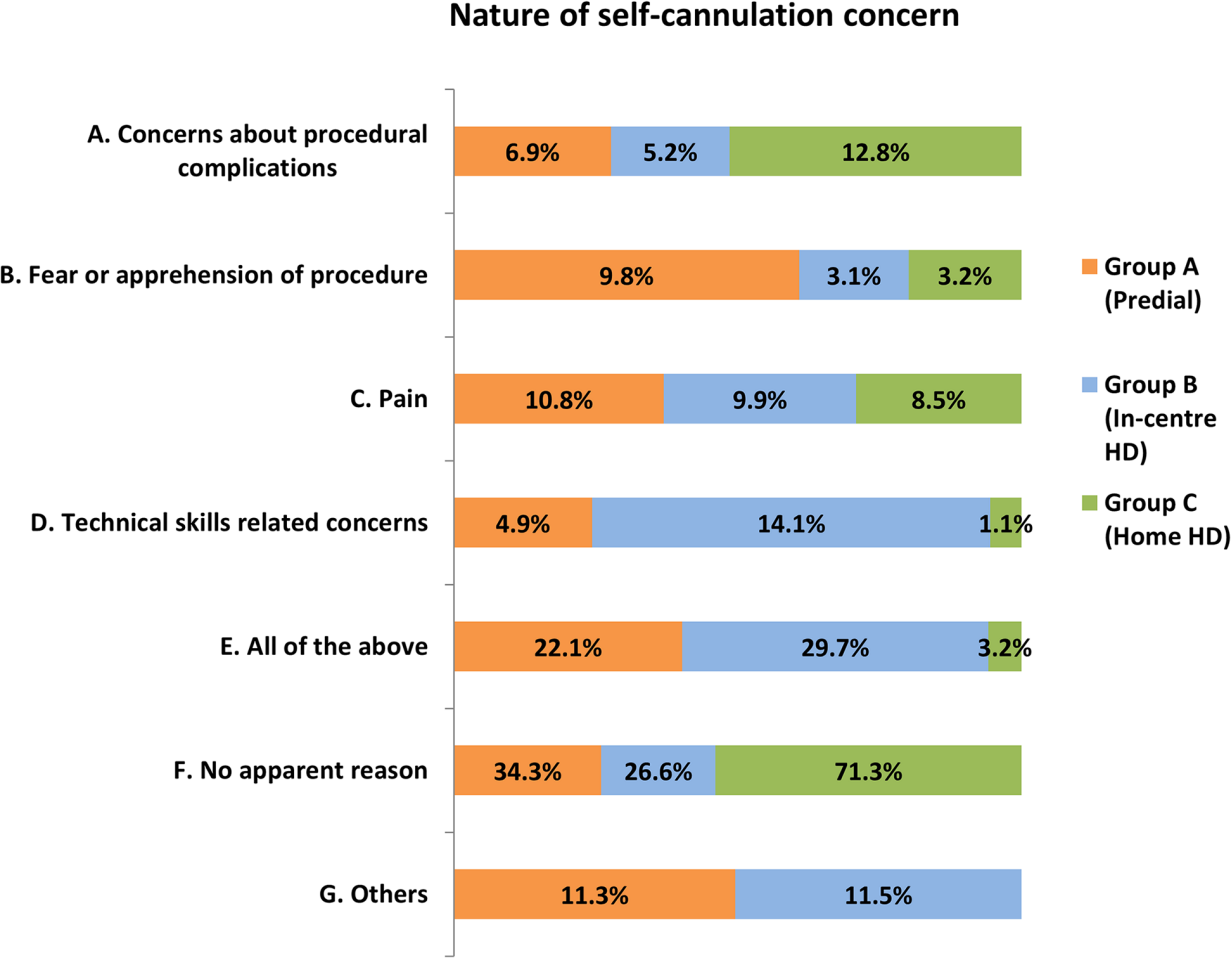
Bar chart depicting the nature of self-cannulation concern amongst patients in the three study groups.

Concerns for self-cannulation were identified by patients in all study groups. Groups responded differently to this question. 30.3% of group B and 22% of group ‘A’ patients were fearful and concerned about all aspects of the procedure. Concerns over procedural complications were significantly higher in the self-care HD group (P<0.05). Pain as a cause for concern was felt equally in all study groups (approximately 10%). Technical-skills related apprehension was identified in 14.4% of in-centre patient responses as against 5% of predialysis and 1% of self-care HD groups’ responses (p<0.05). The ‘others’ category comprised of responses that did not fit into the other pre-defined categories and include reasons such as ‘watching the needle insertion’ and ‘lack of confidence’.

**Fig 3:**
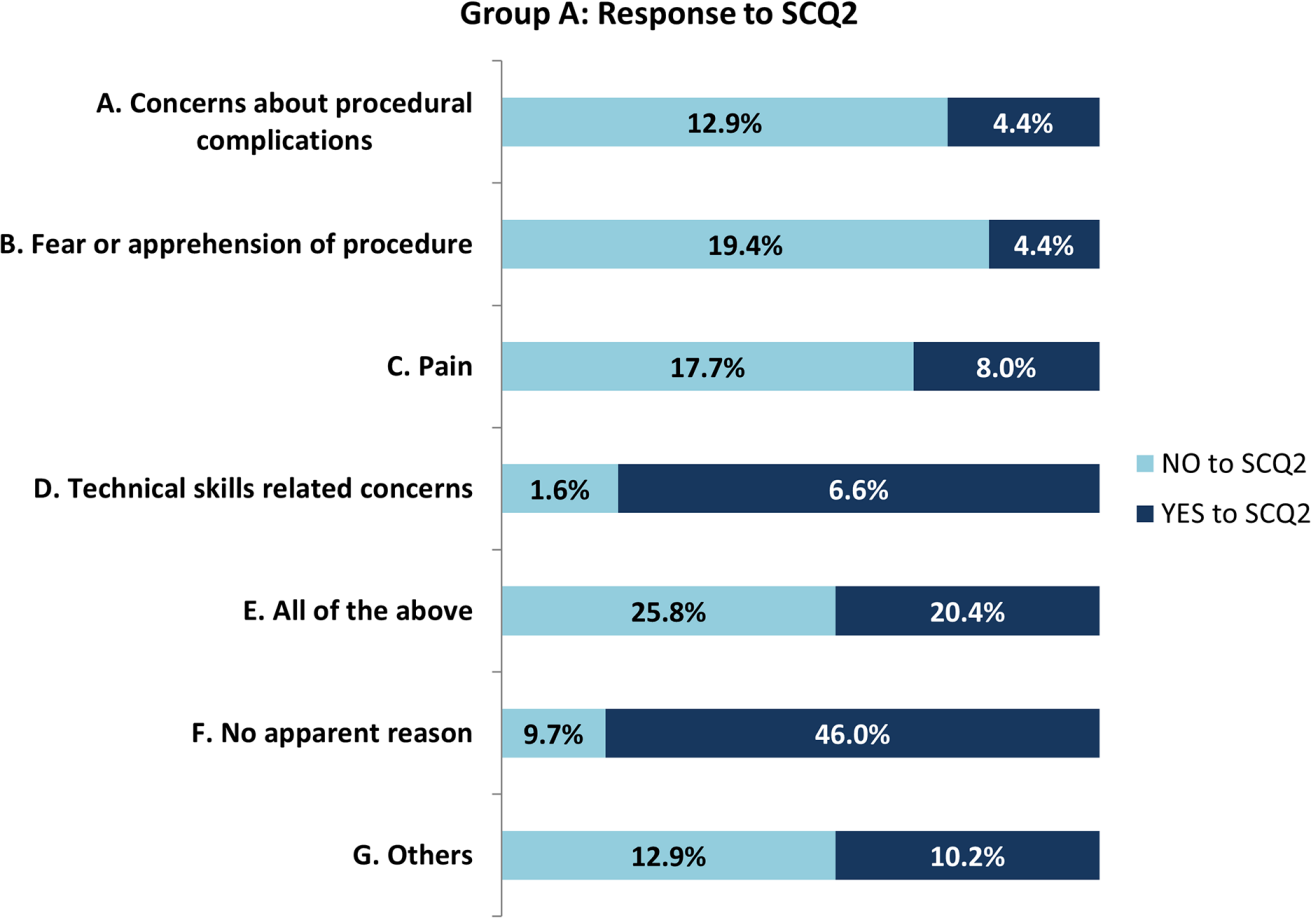
Bar chart depicting reasons for a negative response to ‘perceived ability to self-cannulate’ amongst predialysis (Group A) patients.

Predialysis patients who felt able or unable to self-cannulate identified their reasons (Fig 3). Significant differences in apprehensions between the negative and positive responders to SCQ2 were, pain (p<0.05) and fear of the procedure itself (p<0.05). About 25% of the ‘No’ responders and 20% of the ‘Yes’ responders felt however, that all aspects of SC were bothersome. 9.7% of the predialysis group with negative disposition to SC did not have any specific reason to dismiss self-cannulation for HD. 6.6% of those who would consider SC cited technical skills and ability as an important consideration as against 1.6% of the negative responders.

### Model Building

The response to SCQ2 was dichotomised into ‘Yes (Yes, Yes, with some help) and ‘No (No and Unsure)’. Variables examined in the univariate analysis are shown in Table 4. Significant determinants of ‘yes’ to SC on univariate analysis in predialysis group (group A), were the types of response to SCQ1 and SCQ3 and lower trait anxiety scores. In addition to the responses to SCQ1 and SCQ3, other significant determinants in the HD group (groups B+C) were, age, education, employment, presence of an informal care-giver, history of malignancy, global cognition scores, TMTB scores and Albumin<30g/L (surrogate of physical illness).

**Table 4:** Univariate analysis.

Variable		Predialysis		In-centre and Home HD	
		Odds ratio (95% CI)	p-value	Odds ratio (95% CI)	p-value
Age (per year)		0.98 (0.95, 1.00)	**0.03**	0.97 (0.95, 0.99)	**0.003**
		**Age-controlled analyses below**			
Gender	Male	1 (-)	0.57	1 (-)	0.22
	Female	0.84 (0.47, 1.52)		0.72 (0.43, 1.12)	
Vision	Normal	1 (-)	0.42	1 (-)	**0.08**
	Poor vision	0.75 (0.37, 1.52)		0.53 (0.26, 1.08)	
Education	High school	1 (-)	0.75	1 (-)	**0.002**
	Post high school	1.12 (0.55, 2.27)		2.67 (1.44, 4.95)	
Employment	Retired	1 (-)	0.62	1 (-)	**<0.001**
	Unemployed	1.41 (0.51, 3.94)		0.54 (0.25, 1.17)	
	Self employed	1.39 (0.43, 4.43)		1.22 (0.45, 3.30)	
	Salaried	1.96 (0.72, 5.33)		4.03 (1.55, 10.52)	
Ethnicity	White	1 (-)	0.75	1 (-)	0.45
	Non-white	0.85 (0.31, 2.35)		0.74 (0.35, 1.59)	
Smoking Status	Never smoked	1 (-)	0.54	1 (-)	0.88
	Ex-smoker	1.27 (0.66, 2.45)		1.01 (0.57, 1.77)	
	Current	0.76 (0.33, 1.76)		0.83 (0.39, 1.76)	
Informal Care-giver	Spouse or partner	1 (-)	0.98	1 (-)	**0.001**
	Child	1.32 (0.37, 4.68)		0.46 (0.15, 1.39)	
	Parent	0.97 (0.22, 4.26)		0.11 (0.03, 0.32)	
	Friend, relative, sibling or carer	1.16 (0.10, 13.39)		0.65 (0.24, 1.81)	
	Alone	0.88 (0.44, 1.77)		0.47 (0.25, 0.87)	
Diabetes	No	1 (-)	0.64	1 (-)	0.66
	Type 1	1.16 (0.21, 6.43)		1.76 (0.46, 6.73)	
	Type 2	1.37 (0.71, 2.67)		0.91 (0.48, 1.69)	
Ischaemic Heart Disease	No	1 (-)	0.21	1 (-)	0.45
	Yes	0.63 (0.31, 1.29)		0.81 (0.46, 1.41)	
Heart Failure	No	1 (-)	0.15	1 (-)	0.13
	Yes	0.38 (0.10, 1.41)		0.42 (0.13, 1.30)	
Stroke	No	1 (-)	0.15	1 (-)	0.22
	Yes	3.21 (0.65, 15.93)		0.55 (0.21, 1.44)	
Solid Organ Malignancy	No	1 (-)	0.96	1 (-)	**0.03**
	Yes	1.03 (0.37, 2.85)		2.44 (1.08, 5.48)	
Body Mass Index (per unit increase)		0.98 (0.94, 1.03)	0.48	1.03 (0.99, 1.07)	0.14
History of Peritoneal Dialysis	No	-	N/A*	1 (-)	0.97
	Yes			1.01 (0.60, 1.70)	
History of Renal Transplantation	No	1 (-)	0.63	1 (-)	**0.09**
	Yes	1.49 (0.29, 7.76)		1.65 (0.93, 2.96)	
Opportunity to speak to other HD patients	No	1 (-)	0.32	1 (-)	0.35
	Yes	0.75 (0.42, 1.33)		1.28 (0.76, 2.17)	
BDI in 6 categories (per category increase)—low score to high score		0.91 (0.74, 1.10)	0.32	1.04 (0.89, 1.22)	0.64
Anxiety State in 4 categories (per category increase)—low score to high score		0.88 (0.65, 1.19)	0.42	1.04 (0.81, 1.35)	0.75
Anxiety Trait in 4 categories (per category increase)—low score to high score		0.73 (0.54, 1.00)	**0.05**	1.05 (0.81, 1.36)	0.70
3MS in 5 categories (per category increase)—high score to low score		0.87 (0.62, 1.20)	0.39	0.51 (0.38, 0.69)	**<0.001**
Trail Making Test B (per unit increase)		1.00 (1.00, 1.01)	0.32	0.99 (0.98, 1.00)	**<0.001**
Meta Cognition Questionnaire 1 (metamemory) (per unit increase)		1.01 (0.94, 1.09)	0.77	1.01 (0.95, 1.07)	0.74
Meta Cognition Questionnaire 2 (metaconcentration) (per unit increase)		1.06 (0.95, 1.18)	0.34	0.98 (0.90, 1.05)	0.53
Autonomy Preference Index- Decision Making (per unit increase)		1.00 (0.98, 1.02)	0.79	1.01 (1.00, 1.03)	0.11
Autonomy Preference Index- Information Seeking (per unit increase)		1.00 (0.97, 1.03)	0.94	1.01 (0.99, 1.04)	0.26
Low Haemoglobin (Hb<9g/dl)	No	1 (-)	0.60	1 (-)	0.76
	Yes	1.57 (0.29, 8.42)		0.85 (0.31, 2.37)	
Low Albumin (Alb<30g/L)	No	1 (-)	0.23	1 (-)	**0.009**
	Yes	0.40 (0.09, 1.75)		0.32 (0.14, 0.75)	
SCQ1 (Routine phlebotomy question)	Do not mind	1 (-)	**0.008**	1 (-)	**<0.001**
	Fearful	0.32 (0.11, 0.93)		0.06 (0.02, 0.22)	
	Realise	0.38 (0.18, 0.79)		0.54 (0.29, 0.99)	
SCQ3 (Nature of concern question)	No apparent reason	1 (-)	**<0.001**	1 (-)	**<0.001**
	Concerns about procedural complications	0.13 (0.03, 0.45)		0.51 (0.19, 1.39)	
	Fear or apprehension of procedure	0.07 (0.02, 0.25)		0.08 (0.02, 0.44)	
	Pain	0.14 (0.05, 0.43)		0.15 (0.06, 0.40)	
	Technical skills related concerns	0.26 (0.06, 1.13)		0.18 (0.07, 0.45)	
	All of the above	0.23 (0.09, 0.58)		0.56 (0.28, 1.10)	
	Others	0.09 (0.03, 0.27)		0.11 (0.04, 0.34)	

N/A* Model does not converge due to small numbers of patients with previous PD history

**Fig 4:**
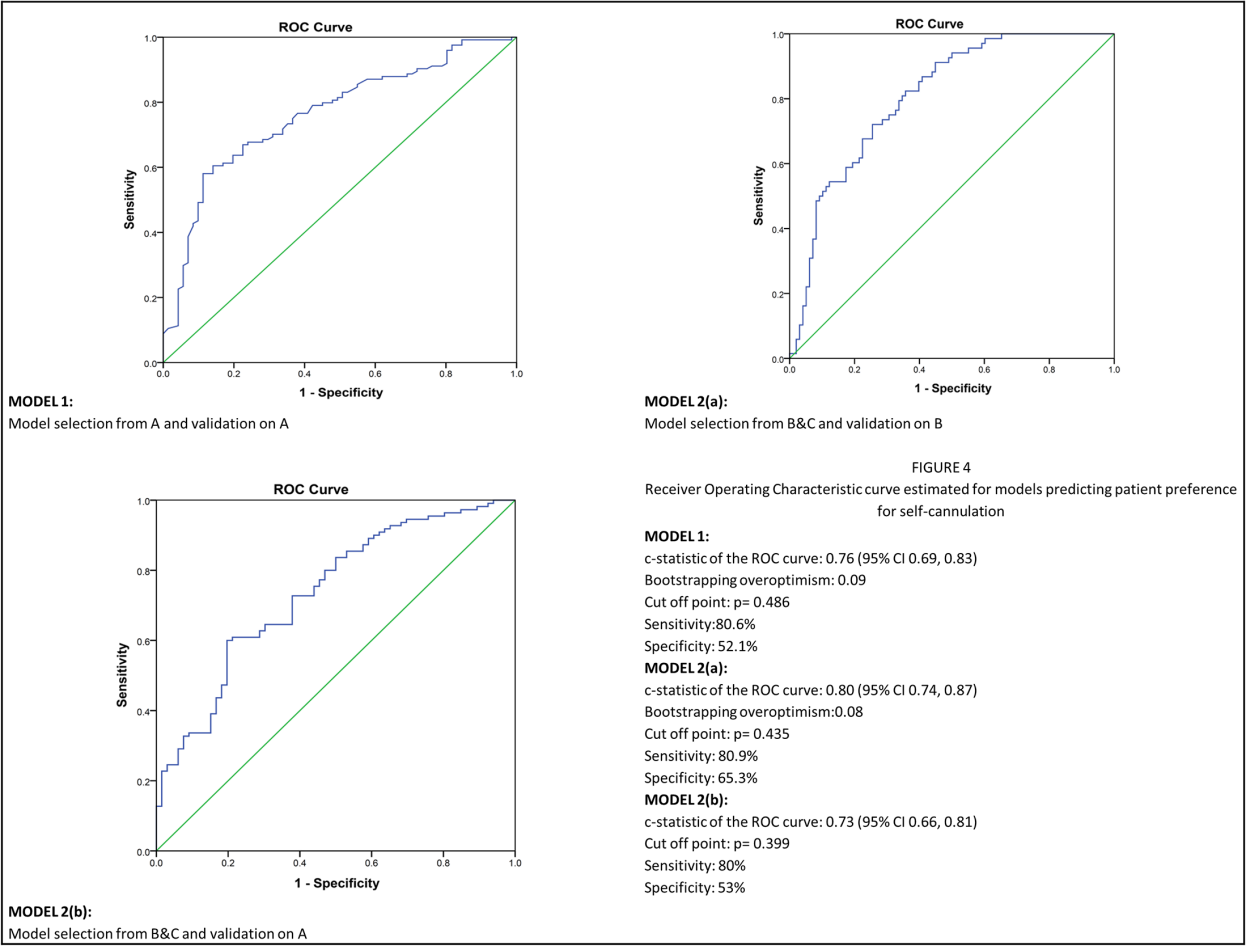
Graphs depicting the Receiver Operating Characteristic (ROC) curves for two models predicting patient preference for self-cannulation.

The final predictors of the outcome (yes to SC) in the predialysis group (Table 5) were lower age, readiness to undertake routine phlebotomy (SCQ1) & the type of SC concern projected (SCQ3). The latter means that compared to the response ‘no apparent reason’, all other category responses are associated with lower odds of responding positively to SC. Significant predictors in the haemodialysis group (Table 5) include lower age, higher education level, higher 3MS category (lower score), absence of low albumin, readiness to undertake phlebotomy and type of SCQ3 response. The latter, in the HD group means that compared to the response ‘no apparent reason’, all other category responses are associated with lower odds of responding positively to SC. Although TMTB was significant in the univariate analysis at 1% significance level, it was not included at multivariable stage, due to high numbers of incomplete datasets (25%). Using the information above, two predictive models were developed to estimate the probability of identifying predialysis and in-centre haemodialysis patients who may consider self-cannulation (Fig 4).

**Table 5:** Multivariable logistic regression analysis.

Model Variables	Predialysis		Haemodialysis	
	Odds Ratio (95% CI)	p-value	Odds Ratio (95% CI)	p-value
**Age (per year)**	0.97 (0.95, 1.00)	**0.05**	0.96 (0.93, 0.99)	**0.01**
**Education (post high school)**	-	**-**	3.31 (1.40, 7.85)	**0.006**
**SCQ1 (Routine phlebotomy question)**		**0.04**		**<0.001**
Do not mind (Reference Category)	1	~	1	~
Fearful	0.42 (0.13, 1.34)	0.14	0.06 (0.01, 0.29)	**<0.001**
Realise	0.34 (0.13, 0.85)	**0.02**	0.52 (0.22, 1.23)	0.14
**SCQ3 (Nature of concern question)**		**<0.001**		**<0.001**
No apparent reason (Reference Category)	1	~	1	~
**A** Concerns about procedural complications	0.13 (0.04, 0.49)	0.003	0.49 (0.15, 1.61)	0.24
**B** Fear or apprehension of procedure	0.14 (0.04, 0.53)	0.004	0.02 (0.00, 0.21)	0.001
**C** Pain	0.16 (0.05, 0.50)	0.002	0.18 (0.05, 0.70)	0.01
**D** Technical skills related concerns	0.29 (0.07, 1.28)	0.10	0.21 (0.08, 0.60)	0.003
**E** All of the above	0.41 (0.14, 1.21)	0.11	1.05 (0.40, 2.76)	0.92
**F** Others	0.09 (0.03, 0.30)	<0.001	0.09 (0.02, 0.36)	<0.001
**3MS category**	-	-	0.59 (0.41, 0.85)	**0.005**
**Low Albumin**	-	-	0.23 (0.07, 0.73)	**0.01**
**Informal Care Giver**	-	-		0.06
Spouse or Partner (Reference Category)	-	-	1	~
Child	-	-	0.62 (0.14, 2.75)	0.53
Parent	-	-	0.18 (0.04, 0.71)	0.01
Friend, Relative, Sibling or Carer	-	-	1.18 (0.30, 4.54)	0.81
Alone	-	-	0.39 (0.17, 0.87)	0.02

Model 1- Derived from group A data (pre-dialysis; n = 202) and validated on ‘A’ data, n = 195. The *c*-statistic from the ROC curve is 0.76 (95% CI 0.69, 0.83).

Model 2(a)—Derived from HD patient data (B+C; n = 246) validating on group B (n = 171). The *c*-statistic from the ROC curve is 0.80 (95% CI 0.74, 0.87).

Model 2(b)—Validating on independent group A (n = 178) data. The *c*-statistic from the ROC curve is 0.73 (95% CI 0.66, 0.81).

The Hosmer-Lemeshow, goodness-of-fit test for ‘A’ has p = 0.58 and for ‘B+C’ on itself has p = 0.12. These p-values come from testing the null hypothesis that the model is correctly specified. So p>0.05 suggests that we do not reject the null hypothesis for either model. Calibration-in-the-large and calibration slope are 0.15 and 0.73 respectively (acceptable) for Model 1; 0.08 and 0.68 respectively (acceptable) for Model 2(a) and 0.26 and 0.64 respectively (suboptimal calibration) for Model 2 (b).

Model 1 is of most interest and the sensitivity and specificity of the model using a probability cut-off score of 0.486 on the ROC co-ordinates, is 80.6% and 52.1% respectively.

The modeling equations are provided in S1 and S2 Files.

## Discussion

This UK multi-centre study provides an in-depth understanding of self-cannulation preferences in ESRD patients.

To our knowledge this is the largest study of its kind to-date, exploring self-cannulation preferences in patients with ESRD. The study has had excellent response rates to the questions on self-cannulation (95%) and associated information from demographic, clinical and psychosocial patient factors(>80%). Furthermore, we have developed models to understand the ‘typology’ of patients who may prefer self-cannulation. A key strength of this study is the way in which some variables have been included for data analyses. Strictly dichotomising variables such as 3MS or BDI, results in loss of information to be ascertained from scores further removed from the cut-off point. Therefore, these variables are included in ordered categories. Not limiting the study to predialysis group that takes decisions on self-cannulation, is very important, so as to incorporate into our understanding, perceptions and characteristics of those who chose to be cannulated (in-centre group) and those self-cannulating (home HD group) for HD. Another notable strength is that, data are generated from five centres which allow generalizability.

From our data, it is apparent that SC is an important barrier to uptake of home or self-care HD. Reassuringly, in many instances, this is a surmountable barrier. In a recent publication by Pipkin et al, a survey of the FHN trial investigators showed that the most commonly perceived barriers to intensive HD included lack of patient motivation, unwillingness to change from in-centre modality, and fear of self-cannulation[15]. Although ‘fear of SC’ is a broad terminology in use, more information needs to be ascertained from patients as to what the ‘fears’ are about. In another qualitative study of hospital HD and nocturnal home HD patients by Cafazzo et al, fear of SC as a deterrent to HHD, is a recurring theme[16]. Population of interest really, is the predialysis cohort, as once established on a therapy; it is rather difficult to electively change modality.

The predialysis cohort is of particular interest, as they engage with the concept of self-care HD, with no practical insight into the process. Understanding reasons for their negative disposition to SC is vital to providing case-specific intervention. The commonly perceived notion in practice, of pain being a significant deterrent for SC is questioned by the observation that the same proportion of individuals in a self-caring cohort, perceive pain, but, other persuasive belief constructs have determined their self-cannulation decision. Although 1 in 5 individuals are likely to find all aspects of SC overbearing, concerns over procedural complications, pain, and technical-skills which may dominate the decision to consider institutional care, are potentially modifiable. It is interesting that about 10% of predialysis patients who may not consider SC, have no specific reason for doing so, and these groups of individuals may be open to influence over their decision, provided other factors are favourable for self-care HD.

About 30% of patients being offered hospital HD feel able to self-cannulate. This suggests a ‘missed opportunity’ to promote self-care and shared-care in dialysis facilities impacting positively on the ever-constrained staff resource on HD units. This is of particular value in situations where, home adaptations are not feasible and all other patient characteristics allow self-care in hospital. This may also result in lesser waiting times before individuals commence HD, and greater patient independence. It is notable that diabetes is not a deterrent to the idea of perceived ability to self-cannulate. This may be due to the fact that patients are able to draw on their experience of subcutaneous insulin injections and may relate at a practical level with the concept of ‘self-needling’. Needless to say, the indirect impact of diabetes through blunting of cognitive abilities from microvascular disease would have adverse effect on the outcome of interest.

We introduce 2 models of ‘self-cannulation preference’. Model 1, from the predialysis group has shown good discrimination and calibration. Model 2, from the two HD groups has shown good discrimination and calibration for the ‘in-centre’ group, but, suboptimal calibration for the predialysis validation group. The latter may be due to change in patient characteristics before and after commencement of HD. This often could be partially remedied through recalibration or structural model revisions (adding new variables). In this area, there is no precedent in the published literature. We have included several clinical and behavioural patient-specific parameters, categorised appropriately. Models have been derived from complete cases with minimal loss of cases to missing information. The predictive accuracies of both models are >70%. Validation and calibration procedures are required for these models to be useful in clinical practice and have been performed. Bootstrap re-sampling is a more effective technique for validating a prediction model than data-splitting. The utility of the models is highest in their respective groups (A on A and B+C on B). Nevertheless, validation of the HD model was also carried out on an independent data set in the ‘predialysis’ group, with a predictive accuracy of 71.3%.

The key challenge lies in the identification and management of the patient who is undecided about self-care for HD solely from SC concerns. The application of the SCQ1 as an initial screening tool to all patients appears to be a good discriminator to understand patient preferences in SC. Predictive modelling in this area of self-care HD is a complement to the clinician’s/nurse’s experience, expertise, and intuition and can fit seamlessly into the clinical process. By offering a systematised way to make clinical decisions and utilize resources, the care planning process can be streamlined. In the context of pre-dialysis care, tools to objectively determine SC preference will help focus resource on patients who need them most, and tailor the nature of intervention to the specific case in question. This data is transferable between members of the multi-disciplinary teams thereby allowing a more standardised predialysis service through better understanding, communication, and cooperation in interdisciplinary teams. The SC decision-aid (currently lacking in the portfolio of dialysis decision choices) may allow patient engagement with SC, even if for ‘trial’ of the procedure. Multifaceted approach to managing such patients is required. These may include behavioural counseling for those concerned for various aspects of the procedure. Innovation in SC training through adoption of virtual, 3-D simulation training may alleviate specific concerns for some. These may also have utility as educational tools in the pre-dialysis phase. The role of expert patients as teachers of SC technique remains to be explored. A further step in targeted SC education and training strategies to a specific subgroup willing to engage and likely to succeed may allow effective resource utilisation to drive better outcomes. The models provide scope for standardisation of care by mitigating subjective biases such as staff perceptions and preferences.

The study has a focus on patient perspective and lacks trainer/caregiver perspective on SC. This may be clinically relevant. A prospectively collected large dataset would be the ideal data from which to derive predictive models. It is important to also externally validate our prediction models on other predialysis and hospital HD patients. That being said, these models (and future improvements to the models) cannot be used as a standalone. These tools are adjuncts to decision-making process by healthcare-providers in order to efficiently manage patients with ESRD and help promote self-care haemodialysis where feasible.

## Conclusion

Self-cannulation in dialysis is a neglected area of research in HD and there is an urgent need to address ‘the elephant in the dialysis room’. There are substantial numbers of ESRD patients who may be able and willing to consider SC. This study provides an insight into the modifiable concerns around self-cannulation.

## Supporting Information

S1 File Model Equation for predicting self-cannulation preference in predialysis patients. (DOCX)

S2 File Model Equation for predicting self-cannulation preference in haemodialysis patients. (DOCX)
