# Supplementary material for: Self-Cannulation for Haemodialysis: Patient Attributes, Clinical Correlates and Self-Cannulation Predilection Models
Source: PLoS One. 2015 May 19;10(5):e0125606. doi: 10.1371/journal.pone.0125606 (PMC4437898; doi:10.1371/journal.pone.0125606)
Supplement: S1 File — (DOCX) [file pone.0125606.s001.docx]

**SUPPLEMENTARY FILE 1: Model Equation for predicting self-cannulation preference in predialysis patients**

**Group A Model: MODEL 1**

ln(p/(1-p)) = 3.60 - 0.03*Age - 0.87*Fearful - 1.09*Realise - 2.01*A - 1.96*B - 1.82*C - 1.24*D - 0.88*E - 2.36*F

where p is the probability of saying yes to the self-cannulation question.

The variables in the above model can be understood as follows:

Age is the patient’s age

There are three categories for SCQ1 (routine phlebotomy question): do not mind, fearful and realise it is important.

Use Fearful = 1 if patient answers the needle insertion for blood test question with fearful, Fearful = 0 otherwise

Use Realise = 1 if patient answers the needle insertion for blood test question with they realise it is important for their well-being, Realise = 0 otherwise

The patient should have answered the needle insertion for blood test question - they do not mind if they are not in one of the other categories. In this case, Fearful = 0 and Realise = 0

SCQ3 (Aspect of Needling that Bothers you most question) has 7 categories:

0: No apparent reason

A: Concerns about procedural complications

B: Fear or apprehension of procedure

C: Pain

D: Technical skills related concerns

E: All of the above

F: Others

If the 0 category is the answer, A-F are all 0 in the model above.

If any of the A-F categories are chosen, substitute 1 for the letter of the patient’s answer in the equation above and substitute 0 for all the other letters.
