# Supplementary material for: Self-Cannulation for Haemodialysis: Patient Attributes, Clinical Correlates and Self-Cannulation Predilection Models
Source: PLoS One. 2015 May 19;10(5):e0125606. doi: 10.1371/journal.pone.0125606 (PMC4437898; doi:10.1371/journal.pone.0125606)
Supplement: S2 File — (DOCX) [file pone.0125606.s002.docx]

**SUPPLEMENTARY FILE 2: Model Equation for predicting self-cannulation preference in haemodialysis patients**

**MODEL 2- Groups B+C**

ln(p/(1-p)) = 4.83 - 0.04*Age + 1.20*Education - 2.75*Fearful - 0.65*Realise - 0.71*A - 3.97*B - 1.69*C - 1.54*D + 0.05*E - 2.38*F - 0.53*3MS category - 1.47*Low Albumin - 0.47*Child - 1.73*Parent + 0.16*FRSC - 0.95*Alone

where p is the probability of saying yes to the self-cannulation question.

The variables in the above model can be understood as follows:

Age is the patient’s age

Use Education = 1 if patient has done post high school education, Education = 0 otherwise

There are three categories for SCQ1 (Needle Question): do not mind, fearful and realise it is important.

Use Fearful = 1 if patient answers the needle insertion for blood test question with fearful, Fearful = 0 otherwise

Use Realise = 1 if patient answers the needle insertion for blood test question with they realise it is important for their well-being, Realise = 0 otherwise

The patient should have answered the needle insertion for blood test question with they do not mind if they are not in one of the other categories. In this case, Fearful = 0 and Realise = 0

SCQ3 (Aspect of Needling that Bothers you most question) has 7 categories:

0: No apparent reason

A: Concerns about procedural complications

B: Fear or apprehension of procedure

C: Pain

D: Technical skills related concerns

E: All of the above

F: Others

If the 0 category is the answer, A-F are all 0 in the model above.

If any of the A-F categories are chosen, substitute 1 for the letter of the patient’s answer in the equation above and substitute 0 for all the other letters.

3MS category is a number between 1 and 5 that corresponds to the 3MS scores as follows:

1: 94-100

2: 86-93

3: 81-85

4: 76-80

5: ≤75

Use Albumin = 1 if the patient has an albumin level less than 30, Albumin = 0 otherwise

There are five categories for Informal Care Giver: spouse or partner, child, parent, alone and a combined category of friend, relative, sibling or carer.

Use Child = 1 if patient’s informal care giver is their child, Child = 0 otherwise

Use Parent = 1 if patient’s informal care giver is their parent, Parent = 0 otherwise

Use FRSC = 1 if patient’s informal care giver is a friend, relative, sibling or carer, FRSC = 0 otherwise

Use Alone = 1 if patient does not have an informal care giver, Alone = 0 otherwise

The patient’s informal care giver should be their spouse or partner if they are not in one of the other categories. In this case, Child = Parent = FRSC = Alone = 0
